# L-Aminoguanidine Induces Imbalance of ROS/RNS Homeostasis and Polyamine Catabolism of Tomato Roots after Short-Term Salt Exposure

**DOI:** 10.3390/antiox12081614

**Published:** 2023-08-15

**Authors:** Ágnes Szepesi, László Bakacsy, Attila Fehér, Henrietta Kovács, Péter Pálfi, Péter Poór, Réka Szőllősi, Orsolya Kinga Gondor, Tibor Janda, Gabriella Szalai, Christian Lindermayr, László Szabados, Laura Zsigmond

**Affiliations:** 1 Department of Plant Biology, Institute of Biology, Faculty of Science and Informatics, University of Szeged, Közép fasor 52, H-6726 Szeged, Hungary; bakacsy@bio.u-szeged.hu (L.B.); feher.attila@brc.hu (A.F.); henrietta.kovacs96@gmail.com (H.K.); palfipeter98@gmail.com (P.P.); poorpeti@bio.u-szeged.hu (P.P.); szoszo@bio.u-szeged.hu (R.S.); 2 Institute of Plant Biology, Biological Research Centre (BRC), Eötvös Loránd Research Network (ELKH), Temesvári krt. 62, H-6726 Szeged, Hungary; szabados.laszlo@brc.hu (L.S.); zsigmond.laura@brc.hu (L.Z.); 3 Centre for Agricultural Research, Eötvös Loránd Research Network (ELKH), Brunszvik u.2., H-2462 Martonvásár, Hungary; gondor.kinga@atk.hu (O.K.G.); janda.tibor@atk.hu (T.J.); szalai.gabriella@atk.hu (G.S.); 4 Institute of Biochemical Plant Pathology, Helmholtz Zentrum Munich, Ingolstädter Landstr. 1, 85764 Neuherberg, Germany; lindermayr@helmholtz-muenchen.de; 5 Institute of Lung Health and Immunity, Comprehensive Pneumology Center, Helmholtz Munich, Max-Lebsche-Platz 31, 81377 Munich, Germany

**Keywords:** salt stress, polyamines, copper amine oxidases, nitric oxide, hydrogen sulfide, L-aminoguanidine, inhibitor, tomato

## Abstract

Polyamine (PA) catabolism mediated by amine oxidases is an important process involved in fine-tuning PA homeostasis and related mechanisms during salt stress. The significance of these amine oxidases in short-term responses to salt stress is, however, not well understood. In the present study, the effects of L-aminoguanidine (AG) on tomato roots treated with short-term salt stress induced by NaCl were studied. AG is usually used as a copper amine oxidase (CuAO or DAO) inhibitor. In our study, other alterations of PA catabolism, such as reduced polyamine oxidase (PAO), were also observed in AG-treated plants. Salt stress led to an increase in the reactive oxygen and nitrogen species in tomato root apices, evidenced by in situ fluorescent staining and an increase in free PA levels. Such alterations were alleviated by AG treatment, showing the possible antioxidant effect of AG in tomato roots exposed to salt stress. PA catabolic enzyme activities decreased, while the imbalance of hydrogen peroxide (H_2_O_2_), nitric oxide (NO), and hydrogen sulfide (H_2_S) concentrations displayed a dependence on stress intensity. These changes suggest that AG-mediated inhibition could dramatically rearrange PA catabolism and related reactive species backgrounds, especially the NO-related mechanisms. More studies are, however, needed to decipher the precise mode of action of AG in plants exposed to stress treatments.

## 1. Introduction

Salt stress is one of the most important abiotic stress factors threatening agriculture and food security globally [1]. Under increasingly challenging climate conditions, more efforts are needed to understand the mechanisms of salt stress and salt tolerance of crop plants [2]. Plants have evolved many different strategies to cope with salinity stress, for example, by synthesizing compounds that alleviate salt stress injury [3].

Polyamines (PAs) are hub molecules in drought and salt stress in plants [4,5]. These essential N-containing molecules occur in different forms in plants, can be free, bound to high-molecular-weight compounds or conjugated to low-molecular-weight ones [6,7]. Many studies have revealed that the accumulation of PAs is a general effect of salt stress on plants, and the extent depends on the plant species, the duration of the stress, or the plant organs affected [8,9,10,11]. PAs are able to reprogram oxidative and nitrosative statuses and the proteome in citrus plants during salt stress [12].

Their involvement in development and abiotic stress response is crucial, as they can interact with and influence other signal molecules, such as nitric oxide (NO) or hydrogen sulfide (H_2_S), both of which are important players in plant abiotic stress signaling [13,14,15].

The biosynthesis of PAs occurs via two pathways: from L-arginine via the enzyme arginine decarboxylase (ADC) or from L-ornithine via the enzyme ornithine decarboxylase (ODC). The function of these enzymes is developmental stage- and organ-dependent in plants during abiotic stress conditions [10].

Besides biosynthesis, PA catabolism is one of the most important processes for fine-tuning the PA levels in salt stress [15,16]. The enzymes involved in catabolic reactions are diamine oxidases (DAOs) and polyamine oxidases (PAOs) [17,18]. Recently, the prominent role of DAOs in the control of plant development and abiotic stress tolerance has been emphasized [19,20]. The precise role of these enzymes in salt stress responses and their interplay with other signaling pathways are, however, not well understood. Fast activation of the genes involved in PA catabolism was shown to happen within one hour after salt treatment [21]. Reduced cytoplasmic PAO activities led to enhanced tolerance to salt and drought stress in *Arabidopsis* by suppressing reactive oxygen species (ROS) production and inducing the expression defense genes [22]. Deregulation of apoplastic PAO could affect tobacco development and influence responses to salt stress [23].

ROS are crucial in plant abiotic stress signaling [24]. Their production, transport, and scavenging by enzymatic and non-enzymatic antioxidants were shown to be tightly connected to various signaling pathways. ROS were shown to influence PA homeostasis, whereas PAs are known to modulate redox homeostasis during salt stress [25]. Spermidine oxidation could induce hydrogen peroxide (H_2_O_2_) production in abiotic stress conditions [26]. Copper amine oxidases (CuAOs) were shown to control NO levels, as the *Arabidopsis cuao* mutant (*AtCuAO*) plants displayed impaired NO production [27,28]. Copper amine oxidase 8 (CuAO8) could regulate the arginine-dependent NO production in *Arabidopsis thaliana* [29]. Nitric oxide (NO) is an important gaseous signal molecule and one of the most crucial reactive nitrogen species (RNS) in plants. NO production is connected to PA homeostasis, as its production from L-arginine is enhanced by PAs [30,31,32]. NO levels could be under feed-back regulation, and NO can also influence abiotic stress responses through the modulation of ROS signals [33] However, there is a gap in our knowledge about the significance of PA catabolism in inducing ROS/RNS production during short-term salt stress in tomatoes.

In order to study the significance of the enzymes of PA catabolism in salt tolerance, different inhibitors which can suppress enzyme activities have been used [34,35]. L-aminoguanidine (AG) is one of those inhibitors which can block the activities of DAOs [34,36]. Efficiency of enzyme inhibition depends on the time of application, the concentration of the inhibitor, and the accompanying stress treatments [34]. Despite being one of the most commonly used inhibitors, controversial effects of AG have been observed. We have observed cultivar-dependent effects of AG treatment on tomato germination [37].

PA metabolism is strongly dependent on the nitrogen (N) balance in plants [38,39]. N metabolism was shown to be regulated by interacting PA and NO signals [40]. Nitrite is not only a N source but is also involved in NO production through a reductive biosynthetic pathway [41]. This process contributes to S-nitrosothiol (SNO) production and subsequent S-nitrosylation of proteins, which is an efficient post-translational modification in plants [42,43,44]. Salinity could induce changes in the S-nitrosylation pattern of mitochondrial proteins in *Pisum sativum* [45]. Additionally, S-nitrosylation or denitrosylation could act as a regulatory mechanism of salt-stress sensing in sunflower seedlings [46].

Our knowledge about the involvement of PAs and their catabolism in short-term salt stress is limited [47], and their contribution to triggering other signaling pathways has not been studied. The objective of this research was to study and give an overview of the short-term effect of salt stress and AG treatment on PA catabolism and related pathways to decipher the significance of PA catabolism and related pathways in the regulation of the fast stress responses of tomato roots.

## 2. Materials and Methods

### 2.1. Plant Material and Growth Conditions

The tomato plants used in this study were *Solanum lycopersicum* Mill. L. cv. Rio Fuego. Seeds were germinated at 26 °C for 3 d in the dark, and the seedlings were subsequently transferred to perlite for 2 weeks. The plants were then placed in a hydroponic culture, as described [8]. The plants were grown for 6 weeks in a controlled environment under 200 µmol m^−2^ s^−1^ photon flux density (F36W/GRO lamps, OSRAM SYLVANIA, Danvers, MA, USA), with a 12/12-h light/dark period, day/night temperatures of 24/22 °C, and relative humidity of 55–60%.

### 2.2. NaCl- and AG-Inhibitor Treatments

Salinity treatment was applied to 6-week-old tomato plants by supplementing the hydroponic solution with 100 and 250 mM NaCl for 1 h in the absence or presence of 1 mM L-aminoguanidine (AG). The concentration of AG was defined to inhibit DAO activity as described [37]. Whole root samples were used for biochemical analysis, whereas root apical tips were used for fluorescent staining. The experiments were repeated three times. In order to avoid the diurnal changes of PA homeostasis, sampling was performed at the same time in all experiments.

### 2.3. Polyamine Catabolic Enzyme Activities

DAO (EC 1.4.3.6) and PAO (EC 1.4.3.4) activities were estimated spectrophotometrically, as described by [26] with some modification. Approximately 200 mg of root tissue was homogenized in liquid N_2_, and 0.6 mL extraction buffer was added to each sample. The extraction buffer contained 0.2 M TRIS (hydroxymethyl)aminomethane (pH 8.0); 10% glycerol; 0.25% Triton X-100; 0.5 mM phenylmethanesulfonyl fluoride (PMSF); 0.01 mM leupeptin in 100 mM potassium phosphate buffer (pH 6.6). The homogenates were left on ice for 20 min and centrifuged for 10 min at 7000× *g* at 4 °C (Eppendorf centrifuge 5424R, Eppendorf GMBH, Hamburg, Germany). A 150 μL aliquot of the supernatant was combined with 600 μL 100 mM potassium phosphate buffer (pH 6.6), and then the reaction was started by adding 22.5 μL of 2-aminobenzaldehyde (from 10 mg/mL stock solution) and 1 M putrescine (Put) for DAO and 1 M spermidine (Spd) for PAO activity measurements. The reaction mixture was incubated for 1.5 h at 37 °C, and the reaction was stopped by adding 50 μL of 20% (*w*/*v*) trichloroacetic acid (TCA). The absorbance was determined at 430 nm (KONTRON, Milan, Italy). The enzyme activity was expressed as specific activity (U g^−1^ FW), where one unit (U) represents the amount of enzyme catalyzing the formation of 1 μmol of Δ^1^-pyrroline min^−1^.

### 2.4. Determination of Free PA Content Using HPLC

Free PA contents were determined as described by [37]. In brief, 200 mg of root was homogenized in 5% perchloric acid. After centrifugation, 2.5 mL of the supernatant was neutralized with 1 mL of 2 M NaOH, and PAs were then derivatized with 10 µL of benzoyl chloride and separated using HPLC. The applied standards were Put, Spd, and spermine (Spm) in the form of hydrochlorides. The results are the means of three independent biological samples expressed in nmol g^−1^ fresh weight^−1^.

### 2.5. RNA Purification and Gene Expression Analyses with Quantitative Real-Time PCR

Total RNA was extracted from 100 mg of tomato root using GeneJET Plant RNA Purification Kit (Thermo Scientific™, K0801 from Thermo Fisher Scientific, Waltham, MA, USA) as recommended by the manufacturer. The isolated RNA was DNase-treated with a TURBO DNA-free™ Kit (Invitrogen by Thermo Fisher Scientific), and first-strand cDNA synthesis of 1 µg of total RNA was carried out with a High-Capacity cDNA Reverse Transcription Kit (Applied Biosystems by Thermo Fisher Scientific), using random hexamers. Real-time PCR was carried out with an ABI 7900 Fast Real-Time System (Applied Biosystems by Thermo Fisher Scientific) with the following protocol: 40 cycles at 95 °C for 15 s, 60 °C for 1 min, using Maxima SYBR Green qPCR Master Mix (Thermo Fisher Scientific). The relative expression levels were normalized to both the *SlEF1* and *SlUBI3* reference genes. The normalized relative transcript levels were calculated according to [48,49] using the 2^−ΔΔCt^ method, where the relative gene expression level of the untreated control was 1. The specific primers for each examined gene are described in Table S1 and related references are cited in the Supplementary Materials.

### 2.6. Hydrogen Peroxide Determination

The H_2_O_2_ levels of the tomato tissues were measured in six-week-old plants using an Amplex Red Hydrogen Peroxide/Peroxidase Assay Kit (Thermo Fisher Scientific, A22188) as recommended by the manufacturer. Approximately 100 mg of fresh plant material was harvested, ground in liquid N_2_, and diluted in 20 mM potassium phosphate buffer (pH 6.5). The homogenates were centrifuged, and the supernatant was used to measure the H_2_O_2_ content. The accumulation of resorufin was determined spectrophotometrically at 560 nm (Thermo Scientific, Multiscan Go Microplate Spectrophotometer). The amount of H_2_O_2_ was calculated using a standard curve.

### 2.7. Superoxide Dismutase Enzyme Activity Measurement

Enzyme extracts were prepared as described by [50]. SOD (EC 1.15.1.1) activity measurement was based on the ability of the enzyme to inhibit the photochemical reduction in p-nitro-blue tetrazolium chloride (Sigma-Aldrich, St. Louis, MO, USA) in the presence of riboflavin in the light. One enzyme unit (U) of SOD represents the amount of enzyme causing a 50% inhibition of p-nitro-blue tetrazolium chloride reduction, and its activity was calculated as U g^–1^ fresh weight.

### 2.8. Histochemical In Situ Detection of Reactive N, O, and S Species

NO production was visualized as described by [51]. NO was visualized in tomato root tips using 4-amino-5-methylamino-2′,7′-difluorofluorescein diacetate (DAF-FM DA) dye. The samples were incubated for 30 min at room temperature in the dark in 10 μM DAF-FM DA dissolved in 10 mM TRIS-HCl buffer (pH 7.4). Superoxide anion (O_2_^•−^ levels were investigated using 10 µM fluorescent DHE (dihydroethidium) [52]. H_2_S determination was visualized by WSP-1 (Washington State Probe-1). Root tips were stained for 40 min in WSP-1 solution, washed three times and examined by a microscope as described by [53]. After staining, the samples were rinsed twice with 10 mM TRIS-HCl buffer (pH 7.4). Fluorescence intensity (pixel intensity) was detected with a Zeiss Axiowert 200M-type fluorescence microscope (Carl Zeiss Inc., Jena, Germany), as described earlier [53]. Filter set 10 (exc.: 450–490, em.:515–565 nm) was used for DAF-FM and WSP-1, and filter set 9 (exc.: 450–490, em.: 515–∞ nm) for DHE [54].

### 2.9. Determination of SNOs and Nitrite Content

Approximately 100 mg of plant material was harvested immediately after the treatments. After freezing in liquid N_2_, the samples were homogenized twice for 10 s using a Silamat S6 tissue homogenizer (Ivoclar Vivadent, Schaan, Liechtenstein) and 1.7–2.0 mm glass beads (Roth). The homogenized root material was extracted in 500 µL 1× phosphate-buffered saline (PBS) and incubated on ice for 10 min, followed by centrifugation for 10 min at 12,435 rpm. The protein content of the plant extract was determined using the Protein Assay Dye Reagent Concentrate (Bio-Rad, Hercules, CA, USA). The quantification of nitrite and SNOs in tomato root tissues was performed using Sievers’ Nitric Oxide Analyzer NOA 280i (GE Water & Process Technologies, Ratingen, Germany) as described by [41]. Endogenous nitrite was reduced to gaseous NO by the injection of root extracts into a reaction vessel containing triiodide solution (28.5 mM I_2_, 66.9 mM KI in 77.7% acetic acid) at 30 °C. For SNO detection, endogenous nitrite was scavenged by adding 5% sulfanilamide (*w*/*v*, in 1 M HCl) at a dilution of 1:9 to the sample before injection into the triiodide solution.

### 2.10. GC-MS Analysis of TCA Cycle Metabolites

After sample collection, 0.1 g portions of the samples were ground in liquid N_2_ and transferred into 2.0 mL safety Eppendorf tubes, containing 30 µL ribitol (1 mg/mL) as an internal standard (ISTD), in 0.5 mL of 60% (*v*/*v*) methanol. The tubes were vortex-mixed for 30 s, then placed in an ultrasound bath for 5 min, vortex-mixed again for 15 s, and centrifuged for 5 min at 10,000 rpm, and the supernatant was collected. The extraction was repeated with 0.5 mL of 60% (*v*/*v*) methanol and again with 0.5 mL of 90% (*v*/*v*) methanol. The supernatant was collected and mixed well. An aliquot (100 µL) was dried in vacuum. For derivatization, methoxyamine hydrochloride dissolved in pyridine (20 mg/mL) was added, incubated at 37 °C for 90 min., then *N*-trimethylsilyl-*N*-methyl trifluoroacetamide was added and incubated for 30 min at the same temperature.

The samples were transferred to vials and injected split-mode into the Shimadzu GCMS-TQ equipped with GC column (Phase: HP-5MS length 30 m; ID 0.25 mm; Film thickness: 0.25 µm). A total of 1 µL was injected into the column at 230 °C, and the transfer line and ion source were 250 °C. The carrier gas was He, and a constant flow rate (1 mL/min) was used. The thermal program was 70 °C for 1 min, which increased to 320 °C at a rate of 7 °C/min, and the high temperature was maintained for 5 min. The Kovats retention index was used to identify the standards, and GCMSSolution 4.16 for Shimadzu was used for data processing. Both the GC analyses and data processing were carried out with GCMSSolution 4.16 for Shimadzu using the Wiley and Nist databases.

### 2.11. Statistical Analysis

The experiments were carried out in three independent biological repetitions. The data are given as the mean ± standard deviation (SD), calculated from the combination of biological repetitions. Two-way analysis of variance (ANOVA) was carried out using GraphPad Prism version 8.0.1.244 for Windows (GraphPad Software, La Jolla, CA, USA), with significance level 0.05 (*p* ≤ 0.05) (interaction analysis can be seen in Table S3). Different letters on the bars denote significant differences (*p* = 0.05) based on Tukey’s post hoc test for multiple comparisons.

## 3. Results

### 3.1. AG Has a Nonspecific Inhibitory Effect on NaCl-Induced Concentration-Dependent Alterations in DAO and PAO Activities of Tomato Roots

**Figure 1:**
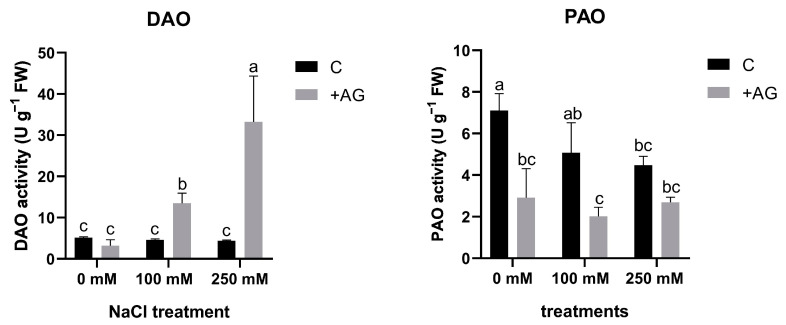
DAO and PAO activities in AG-treated tomato roots with and without salt stress (100 and 250 mM NaCl). Data of enzyme activities are the mean ± SD of three biological replicates. Different letters denote significant differences, two-way ANOVA, following Tukey’s post hoc test *p* = 0.05.

In order to investigate the capacity of AG as a DAO inhibitor, 1 mM AG was added to the nutrient solution, and tomato roots were treated for 1 h. NaCl treatment alone did not affect DAO activity, but a significant reduction in PAO activity was detected by salt stress, which was concentration-dependent (Figure 1). In the presence of AG, DAO activities in tomato roots were enhanced by increasing the concentration of salt (Figure 1).

### 3.2. Free PA Levels Are Reduced by AG Treatment Independent of NaCl Concentration

**Figure 2:**
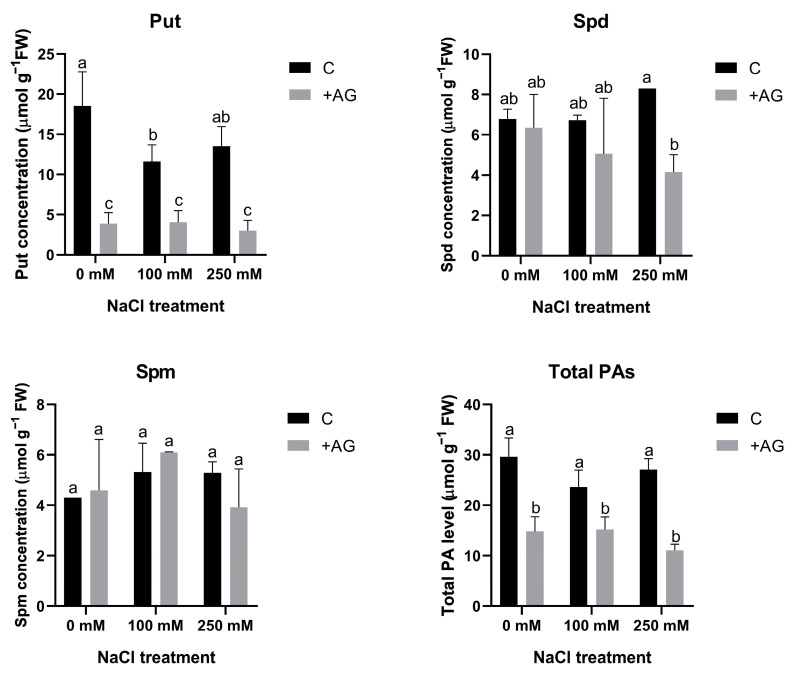
Free polyamine contents of control and AG-treated tomato roots with and without NaCl treatment (100 and 250 mM). Data of PA contents are the mean ± SD of three biological replicates. Different letters denote significant differences, two-way ANOVA, following Tukey’s post hoc test *p* = 0.05.

In order to determine the effect of AG on PA metabolism, free PA concentrations were determined in salt- and AG-treated tomato roots. Total PA contents were lower in AG-treated roots, which were not influenced by salt stress. Put levels were considerably reduced by AG with or without salt treatment. Spd concentration was reduced by AG only in the presence of 250 mM NaCl, whereas Spm contents were not influenced by either AG or salt (Figure 2).

### 3.3. AG Induces Expression of PA Biosynthetic and Catabolic Genes

**Figure 3:**
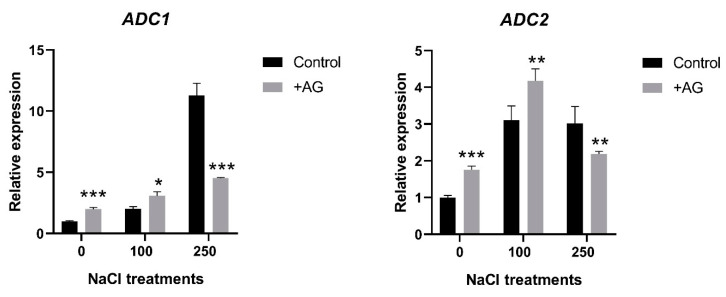
Expression of *ADC* genes of salt- and AG-treated tomato roots. Relative expression levels are shown where 1 represents the untreated control. Data of each bar are the mean ± SD of three biological replicates. Asterisks denote significant differences from untreated control, Student *t* test, * *p*  <  0.05, ** *p*  <  0.01, *** *p*  <  0.001.

In order to study the effect of AG on the activity of genes implicated in the biosynthesis of PAs, transcript levels of *ADC1* and *ADC2* genes encoding arginine decarboxylase, and ODC1 coding for ornithine decarboxylase were determined. Both *ADC1* and *ADC2* were induced by salt, to different degrees. *ADC1* transcript levels were 2 and 12 times higher in 100 mM and 250 mM NaCl-treated roots, respectively. AG treatment slightly enhanced *ADC1* expression in control roots and in the presence of 100 mM NaCl (two and three times, respectively), whereas it reduced *ADC1* expression by 60% compared to its 250 mM NaCl-treated control when stronger salt stress was applied. *ADC2* expression was increased three-fold in salt-treated roots. AG slightly enhanced *ADC2* transcription in control and 100 mM NaCl-treated roots (1.75- and 4.1-fold, respectively), whereas expression of this gene was slightly reduced only by 28% with AG at stronger salt stress (Figure 3). *ODC1* expression was 1.5-fold increased by AG treatment in roots treated with no or 100 mM NaCl, but it was reduced by 250 mM NaCl with or without AG (Figure S1).

**Figure 4:**
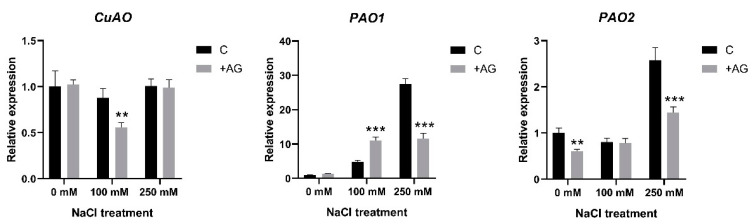
Expression of *CuAO* and *PAO* genes of control and AG-treated tomato roots with and without NaCl treatment. Relative expression levels are shown as in Figure 3. Data of each bar are the mean ± SD of three biological replicates. Asterisks denote significant differences from untreated control, Student *t* test, ** *p* < 0.01, *** *p* < 0.001.

To test whether the altered enzyme activities were derived from the changes in gene expression, we determined the transcript levels of the PA catabolism genes. *CuAO* expression was slightly reduced, only by 30% after AG treatment and only in 100 mM NaCl-treated roots (Figure 4). Considerable variation was observed when the expression of *PAO* genes [21] was tested. Transcript levels of *PAO1* were enhanced by salt in a concentration-dependent manner, displaying 30-fold induction in 250 mM NaCl-treated roots. AG enhanced *PAO1* expression at moderate salt stress but reduced it when high salt stress was applied. Expression of *PAO2* was not affected either by 100 mM NaCl or by AG at this salt concentration, while 250 mM NaCl induced *PAO2* activity 2.5-fold, which was reduced in the presence of AG. Transcript levels of *PAO4* were reduced to half by salt treatments but were not affected by AG (Figure 4 and Figure S2). Collectively, the PAO genes showed higher expression in the 250 mM NaCl-treated plants, which was reduced by AG, except for *PAO4* and *PAO5*.

### 3.4. ROS Levels Are Reduced by AG

**Figure 5:**
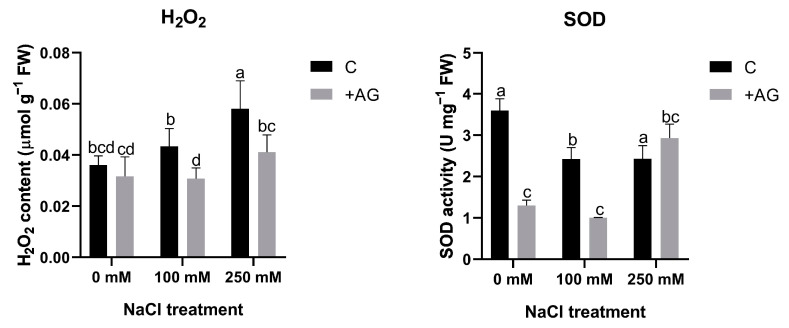
H_2_O_2_ contents and SOD activities of control and AG-treated tomato roots with and without NaCl treatment. Data of each bar are the mean ± SD of three biological replicates. Different letters denote significant differences (two-way ANOVA, Tukey’s post hoc test *p* = 0.05).

To test whether H_2_O_2_ could be altered by reduced PA catabolism, H_2_O_2_ levels were determined in AG- and salt-treated roots. Salt stress enhanced H_2_O_2_ content in a concentration-dependent manner, which was blocked by AG treatment. To further investigate peroxide-derived H_2_O_2_ generation, we subsequently tested the influence of salt and AG on SOD activity. Salt stress slightly reduced SOD, which was inhibited by AG in the absence of salt or when roots were treated by 100 mM NaCl. At higher salt stress, AG had no influence on SOD (Figure 5). These results suggested that SOD activity can be affected by AG in tomato roots, but its influence on H_2_O_2_ content is negligible.

**Figure 6:**
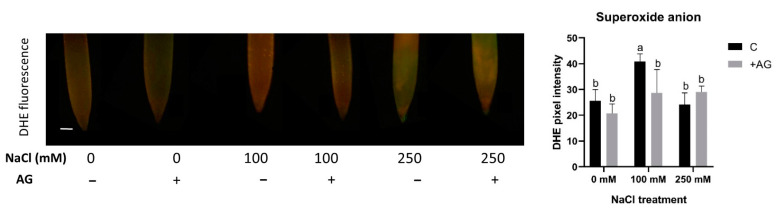
Detection of superoxide anions in control and AG-treated tomato root apices with and without NaCl treatment (100 and 250 mM). Superoxide anion content was measured using a fluorescence probe DHE (dihydroethidium) (scale bar: 100 µm) and expressed as pixel intensity. DHE fluorescence was measured at 500 µm away from the root tips and averaged. Significant reduction occurred in the 100 mM treatment after AG treatment. Bars indicate ±SE of measurements performed with at least 10 seedlings per each treatment, different letters denote significant differences (two-way ANOVA, Tukey’s post hoc test *p* = 0.05).

To test the influence of AG on superoxide production, the accumulation of superoxide anions was tested using in situ DHE assay. Superoxide levels were enhanced by 100 mM but not by 250 mM NaCl. AG had either no or a slightly negative effect on superoxide levels in the root tips (Figure 6).

### 3.5. SNO Increase and Nitrite Reduction Could Be Responsible for Absence of Detectable NO

**Figure 7:**
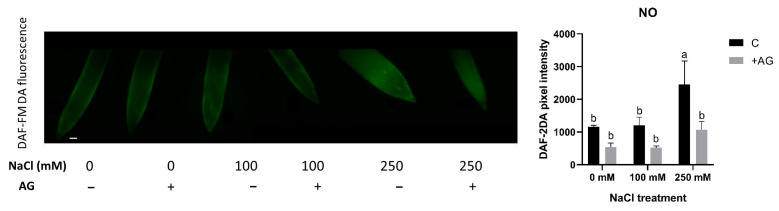
Detection of NO in control and AG-treated tomato root apices with and without NaCl treatment. NO content was detected using a fluorescence probe DAF-FM-DA (scale bar: 100 µm). Quantitative data are expressed as pixel intensity. DAF-FM fluorescence was measured at 500 µm away from the root tips and averaged. Note that AG reduced the NO contents of root tips in all cases. Bars indicate ±SE of measurements performed with at least 10 seedlings per each treatment, different letters denote significant differences (two-way ANOVA, Tukey’s post hoc test *p* = 0.05).

PAs are known to induce NO production in plants. We therefore determined NO levels in salt- and AG-treated tomato root tips using in situ DAF-FM DA fluorescent reaction. While 100 mM NaCl had no effect, 250 mM NaCl treatment led to significantly higher NO accumulation. AG treatment reduced the NO levels only in 250 mM NaCl-treated samples (Figure 7).

**Figure 8:**
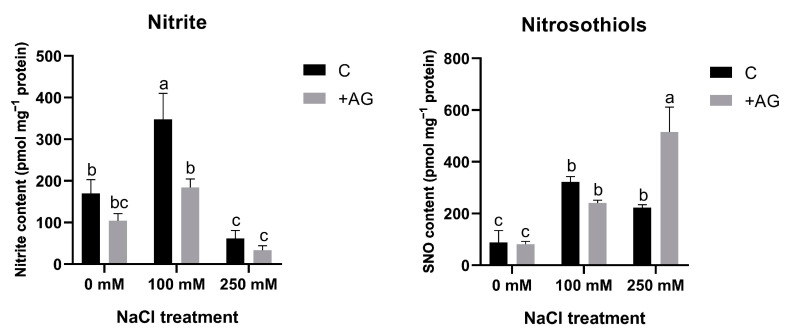
Nitrite and S-nitrosothiol contents of control and AG-treated tomato roots with and without NaCl treatment. Data of each bar are the mean ± SD of three biological replicates. Different letters denote significant differences (two-way ANOVA, Tukey’s post hoc test *p* = 0.05).

NO can be generated from nitrite and nitrosothiols (SNO). In order to compare NO levels with such precursors, nitrite and SNO levels were determined in salt- and AG-treated tomato roots. Nitrite content was higher in roots treated by 100 mM NaCl but was reduced by high salt. Nitrite concentrations were reduced by AG in all samples. SNO levels were enhanced by the NaCl treatments. SNO contents were similar in AG-treated roots in the absence of salt or in the presence of 100 mM NaCl, whereas AG enhanced SNO accumulation in plants stressed by a high concentration of salt (Figure 8).

### 3.6. H_2_S Levels Displayed Different Changes during Salt Stress

**Figure 9:**
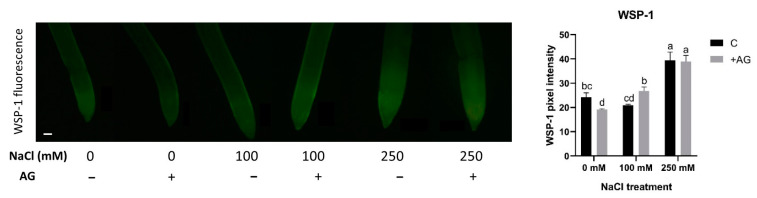
Detection of hydrogen sulfide in control and AG-treated tomato root apices with and without NaCl treatment (100 and 250 mM). Hydrogen sulfide content was measured using a fluorescence probe WSP-1 (Washington State Probe-1) (scale bar: 100 µm) and expressed as pixel intensity. WSP-1 fluorescence was measured at 500 µm away from the root tips and averaged. Bars indicate ±SE of measurements performed with at least 10 seedlings per each treatment, different letters denote significant differences (two-way ANOVA, following Tukey’s post hoc test *p* = 0.05).

Recent studies have suggested that H_2_S could interact with NO and H_2_O_2_ metabolic pathways [55]. Therefore, H_2_S concentrations were measured in salt- and AG-treated root tips, using a specific H_2_S staining method, WSP-1. H_2_S content was not affected by 100 mM NaCl but was increased nearly two-fold by 250 mM NaCl. AG treatment had no or only a slight influence on H_2_S concentrations (Figure 9).

### 3.7. GABA and the TCA Cycle as a Possible Explanation of Decreased PA Levels

Put degradation could result in enhanced GABA production in plants [56]. As reduced PA levels could occur during AG application, we determined the concentrations of GABA and several TCA cycle metabolites in salt- and AG-treated roots (Figure S3). GABA contents slightly increased after 1 h of salt stress, but the difference was not significant. AG reduced the GABA levels in control roots but had no effect on salt-treated roots.

To test possible changes in TCA cycle metabolites, concentrations of citrate, succinate, fumarate, and malate were measured in salt- and AG-treated tomato roots. Moderate salt stress (100 mM NaCl) had only a slight effect on these metabolites, with the exception of fumaric acid, whose concentration was elevated two-fold. Higher salt stress reduced concentrations of succinic, fumaric, and citric acids. AG treatment alone led to reduced amounts of all TCA metabolites tested. In combination with salt stress, AG treatment had no or only a minor influence on the concentrations of these metabolites (Figure S3 and Table S2).

## 4. Discussion

The function of PA catabolism and the enzymes implicated in biosynthesis and catabolism are often studied in relation to specific inhibitors. AG is one of the exogenously applied inhibitor compounds, which are known to block the activity of copper amine oxidase or diamine oxidase. This inhibitor has been demonstrated to decrease DAO enzyme activity, but the effects were found to be different depending on the tested plant species, organ, or the applied dose [36,37]. Short-term salt stress altered PA catabolic responses, similarly to results obtained with tobacco or cucumber, demonstrating a feed-forward ROS amplification loop between ROS-producing NADPH oxidase and the apoplastic PAO [57]. In this study, we tested the specificity of AG when used as a DAO inhibitor in salt-stressed tomato plants. Interestingly, in our experimental conditions, AG was unspecific for DAO and suppressed PAO activity as well (Figure 1). Similar results were reported by Kabala et al. [57]: a 1-h salt treatment did not significantly affect DAO enzyme activity in cucumber, and lower H_2_O_2_ contents were maintained. Although inhibition of DAO enzyme activity was expected to increase PA concentrations, especially Put levels, in our study, reduction in PAO activity by AG treatment correlated with reduced PA levels under both saline and non-saline conditions (Figure 2 and Table S3). To decipher the possible mechanisms of this reduction, the expression of genes which encode PA biosynthesis enzymes such as *ADCs* and *ODC1* and genes which encode DAO and PAO enzymes was studied. We found that salt stress induced ADC expression, especially that of *ADC2*. Interestingly, *ODC1* expression was reduced by 250 mM NaCl and further decreased after AG treatment (Figure S1). Additionally, 100 mM NaCl increased *ADC* and *ODC* expression, which was further enhanced by AG treatment, suggesting that PA levels were increased in these plants (Figure 3). ADC and ODC could compensate for each other’s activity, as revealed by [58]. Meanwhile, 250 mM NaCl was shown to reduce PA content. The expression of genes implicated in PA catabolism in response to salt stress [21] was also tested. *PAO1* of tomato is similar to the *Arabidopsis* AtPAO1, which belongs to Clade 1, and is localized in the cytoplasm. *PAO2*, *PAO4*, and *PAO5* are in Clade 4, showing peroxisome localization. *PAO2* was reported to contribute to ABA-mediated plant developmental processes in *Arabidopsis* [59]. Transcript levels of *PAO4* were reduced to half in salt-treated plants, whereas *PAO5* expression was reduced by 100 mM NaCl but increased in higher salt-stress conditions (Figure S2). *PAO5* expression was previously shown to be unchanged by salt stress, showing a role in xylem differentiation [60]. To explain the differences between these genes, further studies are needed.

Salinity strongly affects ROS signaling in plants [61] by involving the catabolic processes of PAs. PA catabolism could induce H_2_O_2_ production in the roots of tomato plants exposed to salt stress [35]. AG did not change H_2_O_2_ levels compared to the control conditions, providing evidence that AG alone did not induce any additional stress in these plants (Figure 5). AG could alleviate salt-induced H_2_O_2_ accumulation, suggesting that AG might have antioxidant features similar to those reported in soybean [34,36]. To decipher the contribution of other potential biosynthetic processes to H_2_O_2_ production, SOD enzyme activity was measured in salt- and AG-treated roots. SOD activity was reduced by AG in control and 100 mM NaCl-treated roots, but not in the 250 mM NaCl-treated ones. To examine the possible contribution of stress-dependent superoxide production to H_2_O_2_ accumulation, superoxide levels were also measured. AG could reduce superoxide levels in roots subjected to mild salt stress, but it had no significant effect on superoxide production in control or 250 mM NaCl-treated roots (Figure 6). These results are consistent with [57], where short-term salt stress induced slight changes in H_2_O_2_ and SOD in cucumber. It may arise that other antioxidant defense mechanisms could play a role in alleviating oxidative stress during short-term conditions.

AG was suggested as functioning as an irreversible NOS (iNOS) inhibitor widely used for reducing NO production in plants [62]. PAs could be produced from L-arginine, affecting oxidative NO production in plants [63]. In our experimental system, AG reduced NO levels independently of salt stress (Figure 7). A similar effect of AG has been reported in [34], which agrees with other studies. Treatment with 250 mM NaCl could provoke nitrosative stress by inducing reductive or oxidative pathways for NO production, potentially generating reactive nitrogen species for tomatoes, which could be alleviated by AG. To confirm the source of these increased NO levels, we investigated the reductive pathway of NO production. Nitrite could be a source of NO [41,64] in plants. PAs were shown to modulate NR activity in wheat leaves, affecting NO production [65]. Moderate salt stress could enhance nitrite levels, whereas high salinity reduced them, and they were further reduced by AG. SNO is like a stored form of NO. Salinity induced SNO accumulation, which was further increased in 250 mM NaCl-treated tomato roots (Figure 8). There is some evidence for the involvement of SNOs and S-nitrosylation in salinity stress, but these need to be further investigated [66].

Recent results support the role of H_2_S as a signal molecule in salt-stress responses in plants [14,67,68]. Its connection to other signal molecules, such as NO or H_2_O_2_, is almost unknown [69,70]. An early study suggested that NO and H_2_S could interact with each other and regulate each other’s level; however, some other evidence indicates an inhibiting effect of H_2_S on NO production [70]. Additionally, it is known that H_2_S treatment could induce an increase in the expression of genes involved in PA biosynthesis during salt stress [70]. Our results revealed that 250 mM NaCl was able to increase the H_2_S levels, but AG had only a minor influence on H_2_S accumulation in tomato roots (Figure 9). These results confirm that H_2_S production depends on the stress intensity in short-term salt treatment.

To provide further evidence about the decreased PA levels, we also checked the other degradation routes from Put to GABA, which could connect with the TCA cycle as an alternative GABA shunt, contributing to the N balance in plants [71]. Our experiments, however, did not demonstrate any GABA increase or other shift in TCA cycle metabolites by metabolomic analysis (Figure S3).

**Figure 10:**
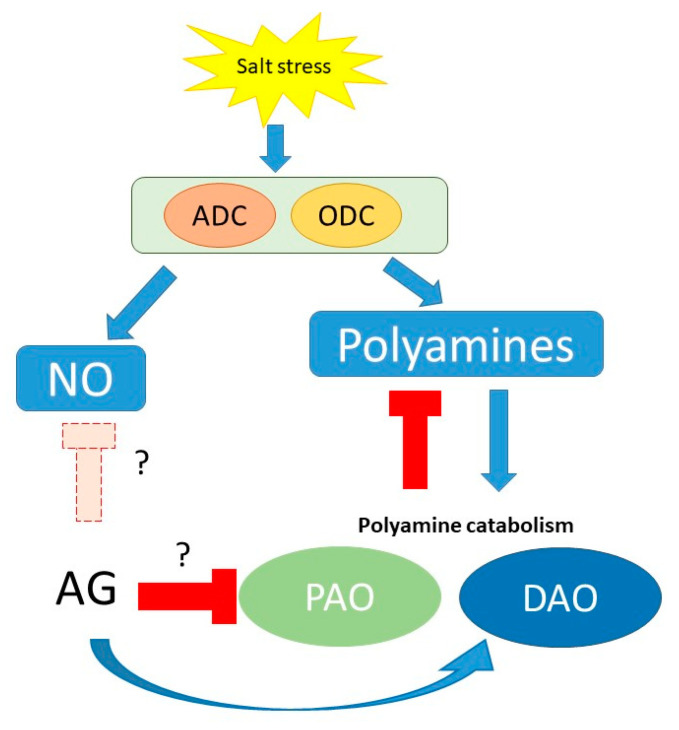
Schematic presentation of potential mechanisms of the effect of AG on PA metabolism and related pathways during short-term salt-stress response to different NaCl concentrations (100 and 250 mM). Question marks indicate the questionable functions of AG during stress, as it can act as an inhibitor of NO synthesis or could induce the expression of *PAO* genes; however, these functions need to be studied.

As a conclusion, our results confirm AG as a nonspecific inhibitor of PA catabolism in short-term conditions, with implications in ROS and NO signaling (summarized in Figure 10 and Figure S4).

Understanding the role of PA catabolism during the early responses of plants to salinity stress could fill the gap in our knowledge about the connection between salt sensing and PA homeostasis, focusing on the interplay between other signal pathways [72]. Precise modes of action of PA catabolism and the related signal pathways need to be deciphered by further studies.

## Data Availability

Not applicable.

## Supplementary Materials

The following supporting information can be downloaded at https://www.mdpi.com/article/10.3390/antiox12081614/s1, Table S1: Primer list of used primers in this study; Figure S1: Expressions of ODC1 gene of control and AG-treated tomato roots with and without NaCl treatment (100 and 250 mM); Figure S2: Expressions of PAO4 and PAO5 genes of control and AG-treated tomato roots with and without NaCl treatment (100 and 250 mM); Figure S3: Heat map of some important TCA cycle metabolite contents of control and AG-treated tomato roots with and without NaCl treatment (100 and 250 mM); Table S2: Levels of some important TCA cycle metabolite contents of control and AG-treated tomato roots with and without NaCl treatment (100 and 250 mM); Table S3: P values for AG and NaCl treatments in case of different parameters; Figure S4: Representative summary of AG treatment and salt stress on tomato roots exposed to NaCl at 100 and 250 mM concentration. References [21,73,74,75,76] are cited in the supplementary materials.
