# Supplementary material for: L-Aminoguanidine Induces Imbalance of ROS/RNS Homeostasis and Polyamine Catabolism of Tomato Roots after Short-Term Salt Exposure
Source: Antioxidants (Basel). 2023 Aug 15;12(8):1614. doi: 10.3390/antiox12081614 (PMC10451491; doi:10.3390/antiox12081614)
Supplement: Supplementary file 1 [file antioxidants-12-01614-s001.zip › antioxidants-2415963-supplementary.pdf]

Additional files:

Table S1. Primer list of used primers in this study.

Sequence (5'-3')	Primer	Target Gene	NCBI Accession	References
TATGCTCACGAGCGAAGAGG	SlPAO1-F	*SlPAO1*	XP_004229651	[1]
GCATTTTCCATGTGCTGCCA	SlPAO1-R	*SlPAO1*	XP_004229651	[1]
TGCTGCAGATGCTGATTCCA	SlPAO2-F	*SlPAO2*	XP_004243630	[1]
AGGACGATATCCCCGAACCA	SlPAO2-R	*SlPAO2*	XP_004243630	[1]
CGGCGTTTTTGTTCACGGAT	SlPAO4-F	*SlPAO4*	XP_004232664	[1]
CCTACCTAAGCGGGGTGGTA	SlPAO4-R	*SlPAO4*	XP_004232664	[1]
CCTGATGCACCTGAACCTGT	SlPAO5-F	*SlPAO5*	XP_004234492	[1]
GGAATCCGGATCTGTTCCCC	SlPAO5-R	*SlPAO5*	XP_004234492	[1]
GCTTATGTTATGACCGCAATCAGT	SlADC1-F	*SlADC1*	Solyc01g110440.4	[2]
TACCCCCGTAAGGAGGCGAT	SlADC1-R	*SlADC1*	Solyc01g110440.4	[2]
TCGGCGGACTCCATAACCTA	SlADC2-F	*SlADC2*	Solyc10g054440.2	[2]
TTACAGCGAAGCTGTGAGGG	SlADC2-R	*SlADC2*	Solyc10g054440.2	[2]
TCCACGACTTCCCTGAGCTA	SlODC1-F	*SlODC1*	Solyc04g082030.1	[2]
AAAGTGAACGCTGTTTCGGC	SlODC1-R	*SlODC1*	Solyc04g082030.1	[2]
TACGGACCAAGTTGTGACTCC	SlODC3-F	*SlODC3*	Solyc03g098310.1	[2]
CTCAGGGAATTGTATGTCAATGGC	SlODC3-R	*SlODC3*	Solyc03g098310.1	[2]
GGGCTGCTGGCTTGTTTG	SlCuAO-F	*SlCuAO*	Solyc08g079430.2.1	[3]
TGATGATACTGTTGGCATTATTGG	SlCuAO-R	*SlCuAO*	Solyc08g079430.2.1	[3]
GGAACTTGAGAAGGAGCCTAAG	SlEF1α-F	*elongation factor 1-α*	NM_001247106	[4]
CAACACCAACAGCAACAGTCT	SlEF1α-R	*elongation factor 1-α*	NM_001247106	[4]
CCAAGATCCAGGACAAGGAA	SlUBI3-F	*ubiquitin 3*	NM_001346406	[5]
AAATCAAACGCTGCTGGTCT	SlUBI3-R	*ubiquitin 3*	NM_001346406	[5]

References

1. Hao, Y.; Huang, B.; Jia, D.; Mann, T.; Jiang, X.; Qiu, Y.; Niitsu, M.; Berberich, T.; Kusano, K.; Liu, T. Identification of seven polyamine oxidase genes in tomato (*Solanum lycopersicum* L.) and their expression profiles under physiological and various stress conditions. *J. Plant Physiol.* **2018**, 228, 1–11.
2. Liu, T.; Huang, B.; Chen, L.; Xian, Z.; Song, S.; Chen, R.; Hao, Y. Genome-wide identification, phylogenetic analysis, and expression profiling of polyamine synthesis gene family members in tomato. *Gene* **2018**, *661*, 1–10. https://doi.org/10.1016/j.gene.2018.03.084.
3. Upadhyay, R.K.; Fatima, T.; Handa, A.K.; Mattoo, A.K. Polyamines and Their Biosynthesis/Catabolism Genes Are Differentially Modulated in Response to Heat Versus Cold Stress in Tomato Leaves (*Solanum lycopersicum* L.). *Cells* **2020**, *9*, 1749. https://doi.org/10.3390/cells9081749.
4. Ma, N.; Feng, H.; Meng, X.; Li, D.; Yang, D.; Wu, C.; Meng, Q. Overexpression of tomato SlNAC1transcription factor alters fruit pigmentation and softening. *BMC Plant Biol.* **2014**, *14*, 1–14. https://doi.org/10.1186/s12870-014-0351-y.
5. Poyatos-Pertíñez, S.; Quinet, M.; Ortíz-Atienza, A.; Yuste-Lisbona, F.J.; Pons, C.; Giménez, E.; Angosto, T.; Granell, A.; Capel, J.; Lozano, R. A Factor Linking Floral Organ Identity and Growth Revealed by Characterization of the Tomato Mutant unfinished flower development (ufd). *Front. Plant Sci.* **2016**, *7*, 1648. https://doi.org/10.3389/fpls.2016.01648.

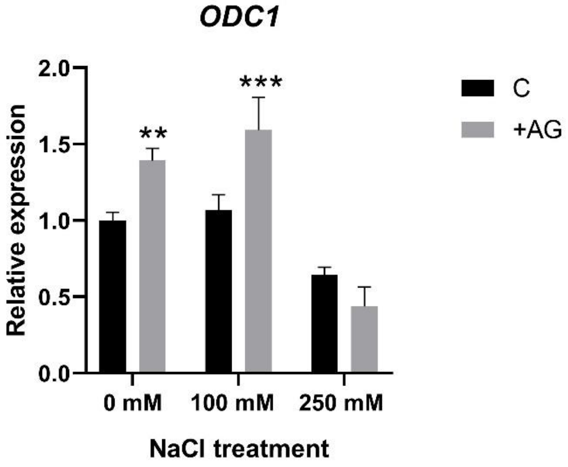

Figure S1. Expressions of *ODC1* gene of control and AG-treated tomato roots with and without NaCl treatment (100 and 250 mM). Relative expression levels were normalized to *SlEF1* and *SlUBI3* reference genes. Data of each bar are the mean ± SD of at least three biological replicates, n = 3. Asterisks denote significant differences from untreated control, Student *t* test, ** $p < 0.01$, *** $p < 0.001$.

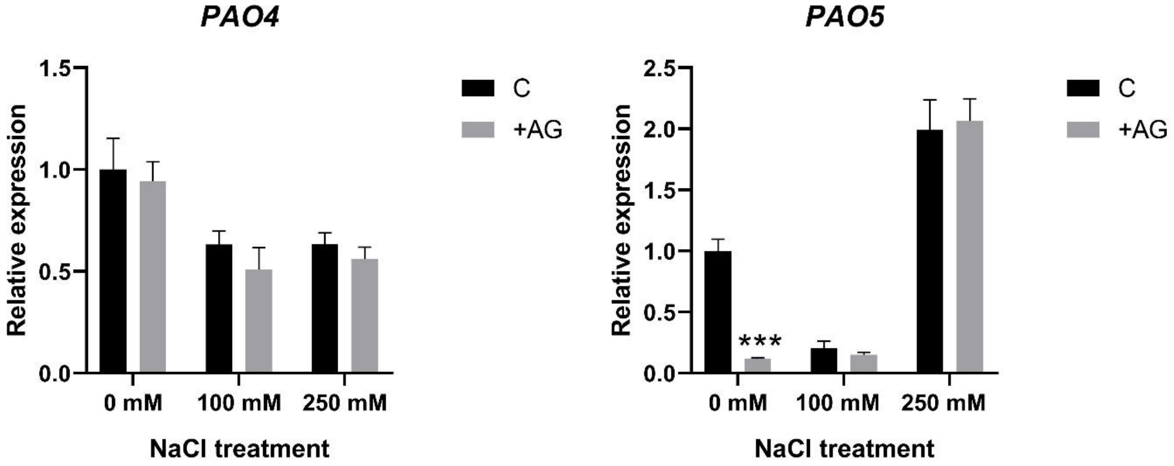

Figure S2. Expressions of *PAO4* and *PAO5* genes of control and AG-treated tomato roots with and without NaCl treatment (100 and 250 mM). Relative expression levels were normalized to *SlEF1* and *SlUBI3* reference genes. Data of each bar are the mean ± SD of at least three biological replicates, n = 3. Asterisks denote significant differences from untreated control, Student *t* test, *** $p < 0.001$.

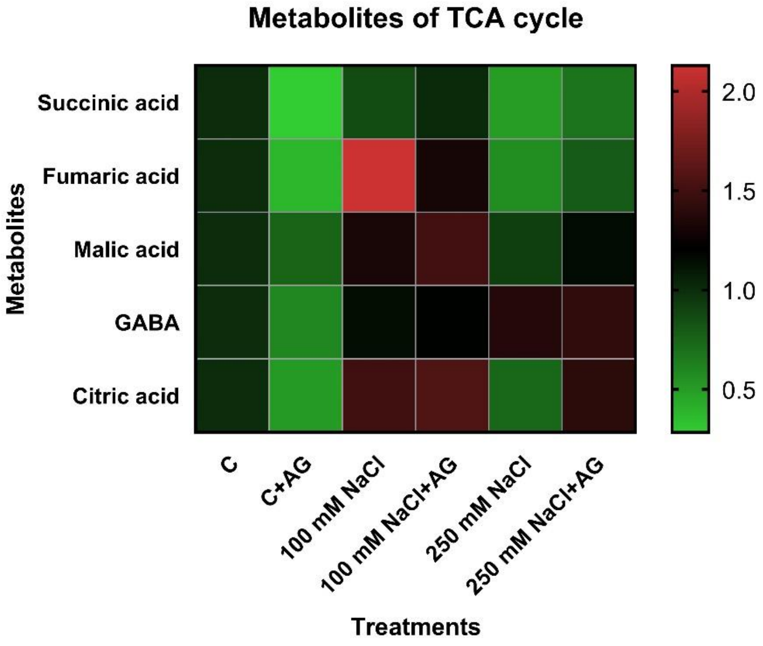

Figure S3. Heat map of some important TCA cycle metabolite contents of control and AG-treated tomato roots with and without NaCl treatment (100 and 250 mM). All data were normalized for control untreated root samples.

Table S2. Levels of some important TCA cycle metabolite contents of control and AG-treated tomato roots with and without NaCl treatment (100 and 250 mM). Different letters denote significant differences (two-way ANOVA, Tukey's post hoc test $p = 0.05$).

Treatments	Succinic acid	Fumaric acid	Malic acid	GABA	Citric acid
Control	29.39±17.19a	6.48±3.44ab	284.18±138.98a	195.36±57.99ab	59.53±28.96a
C+AG	8.32±6.00b	2.43±0.89b	212.17±52.43a	115.87±34.87b	30.34±21.82a
100 mM NaCl	25.37±6.01ab	13.79±8.67a	373.48±156.94a	222.75±81.65ab	88.73±72.84a
100 mM NaCl+AG	29.75±10.75a	8.47±8.04ab	427.22±207.13a	233.67±70.40a	92.77±42.49a
250 mM NaCl	14.46±9.87ab	3.67±2.80b	260.85±145.81a	265.43±72.15a	43.89±9.74a
250 mM NaCl+AG	20.50±6.63ab	5.14±2.64ab	326.69±117.86a	278.01±33.70a	83.5±25.39a

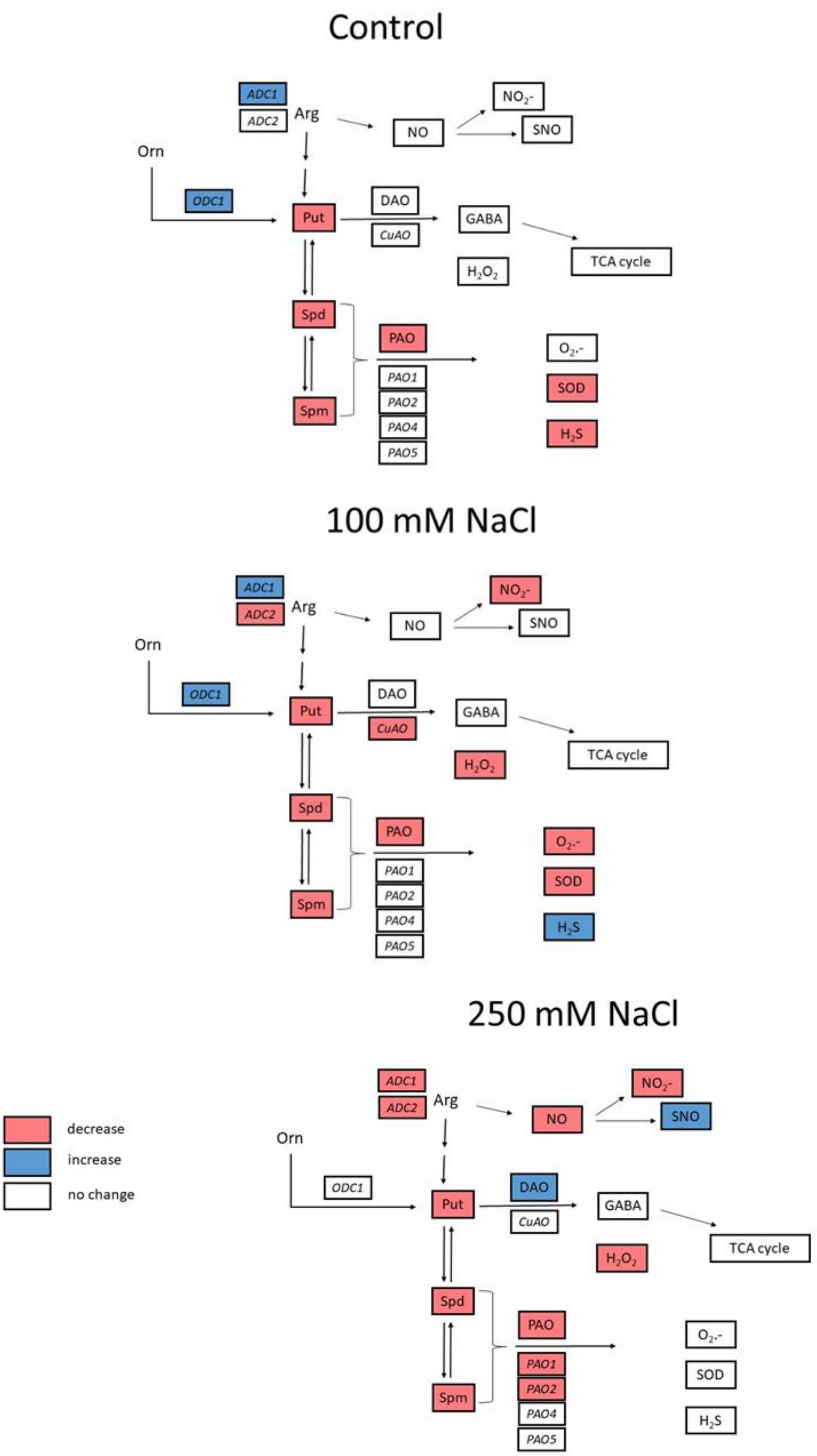

Figure S4. Representative summary of AG treatment and salt stress on tomato roots exposed to NaCl at 100 and 250 mM concentration. Abbreviations in this diagram: *ADC1*—arginine decarboxylase 1; *ADC2*—arginine decarboxylase 2; Arg—L-arginine; *ODC1*—ornithine decarboxylase 1; Orn—L-ornithine; NO—nitric oxide; $NO_2^{\bullet-}$—nitrite; SNO—S-nitrosothiols; Put—putrescine; DAO—diamine oxidase or copper amine oxidase; CuAO—copper amine oxidase; GABA—gamma amino butyric acid; H_2O_2—hydrogen peroxide, TCA cycle- tricarboxylic acid cycle; Spd—spermidine; Spm—spermine; PAO-polyamine oxidase; *PAO1*—polyamine oxidase 1; *PAO2*—polyamine oxidase 2; *PAO4*—polyamine oxidase 4; *PAO5*—polyamine oxidase 5; $O_2^{\bullet-}$—superoxide anion; SOD—superoxide dismutase; H_2S—hydrogen sulfide.

Table S3. P values for AG and NaCl treatments in case of different parameters

Parameter	AG Treatment *p* Value	NaCl Treatment *p* Value	Interaction *p* Value	Summary
DAO<br>Figure 1.	1.65E-04	5.24E-04	3.48E-04	At the 0.05 level, the population means of AG treatment are significantly different.<br>At the 0.05 level, the population means of NaCl treatment are significantly different.<br>At the 0.05 level, the interaction between AG treatment and NaCl treatment is significant.
PAO<br>Figure 1.	1.70E-05	0.028	0.121	At the 0.05 level, the population means of AG treatment are significantly different.<br>At the 0.05 level, the population means of NaCl treatment are significantly different.<br>At the 0.05 level, the interaction between AG treatment and NaCl treatment is not significant.
Put<br>Figure 2.	4.89E-07	0.060	0.069	At the 0.05 level, the population means of AG treatment are significantly different.<br>At the 0.05 level, the population means of NaCl treatment are not significantly different.<br>At the 0.05 level, the interaction between AG treatment and NaCl treatment is not significant.
Spd<br>Figure 2.	0.007	0.702	0.099	At the 0.05 level, the population means of AG treatment are significantly different.<br>At the 0.05 level, the population means of NaCl treatment are not significantly different.<br>At the 0.05 level, the interaction between AG treatment and NaCl treatment is not significant.
Spm<br>Figure 2.	0.848	0.159	0.273	At the 0.05 level, the population means of AG treatment are not significantly different.<br>At the 0.05 level, the population means of NaCl treatment are not significantly different.<br>At the 0.05 level, the interaction between AG treatment and NaCl treatment is not significant.
Total PAs<br>Figure 2.	3.44E-07	0.140	0.076	At the 0.05 level, the population means of AG treatment are significantly different.<br>At the 0.05 level, the population means of NaCl treatment are not significantly different.<br>At the 0.05 level, the interaction between AG treatment and NaCl treatment is not significant.
H_2O_2<br>Figure 6.	9.06E-08	7.03E-09	0.023	At the 0.05 level, the population means of AG treatment are significantly different.<br>At the 0.05 level, the population means of NaCl treatment are significantly different.<br>At the 0.05 level, the interaction between AG treatment and NaCl treatment is significant.
SOD<br>Figure 6.	1.22E-06	7.10E-05	2.06E-06	At the 0.05 level, the population means of AG treatment are significantly different.<br>At the 0.05 level, the population means of NaCl treatment are significantly different.<br>At the 0.05 level, the interaction between AG treatment and NaCl treatment is significant.

DHE<br>Figure 7.	0.021	9.77E-06	9.72E-04	At the 0.05 level, the population means of AG treatment are significantly different.<br>At the 0.05 level, the population means of NaCl treatment are significantly different.<br>At the 0.05 level, the interaction between AG treatment and NaCl treatment is significant.
NO<br>Figure 8.	1.03E-04	5.89E-04	0.129	At the 0.05 level, the population means of AG treatment are significantly different.<br>At the 0.05 level, the population means of NaCl treatment are significantly different.<br>At the 0.05 level, the interaction between AG treatment and NaCl treatment is not significant.
Nitrite<br>Figure 9.	1.08E-04	2.48E-07	0.009	At the 0.05 level, the population means of AG treatment are significantly different.<br>At the 0.05 level, the population means of NaCl treatment are significantly different.<br>At the 0.05 level, the interaction between AG treatment and NaCl treatment is significant.
Nitrosothiols<br>Figure 9.	0.007	4.63E-07	2.65E-05	At the 0.05 level, the population means of AG treatment are significantly different.<br>At the 0.05 level, the population means of NaCl treatment are significantly different.<br>At the 0.05 level, the interaction between AG treatment and NaCl treatment is significant.
WSP-1<br>Figure 10.	0.807	0	1.09E-06	At the 0.05 level, the population means of AG treatment are not significantly different.<br>At the 0.05 level, the population means of NaCl treatment are significantly different.<br>At the 0.05 level, the interaction between AG treatment and NaCl treatment is significant.
